# Barriers in recognising, diagnosing and managing depressive and anxiety disorders as experienced by Family Physicians; a focus group study

**DOI:** 10.1186/1471-2296-10-52

**Published:** 2009-07-20

**Authors:** Eric van Rijswijk, Hein van Hout, Eloy van de Lisdonk, Frans Zitman, Chris van Weel

**Affiliations:** 1 Department of Family Medicine, University Medical Centre St Radboud, PO box 9101, 6500 HB Nijmegen, the Nederlands; 2 Department of Family Medicine EMGO, VU University Medical Centre, Van der Boechorststraat 7, 1081 BT Amsterdam, the Netherlands; 3 Department of Psychiatry, Leiden University Medical Centre, PO Box 9600, 2300 RC Leiden, the Netherlands

## Abstract

**Background:**

The recognition and treatment of depressive- and anxiety disorders is not always in line with current standards. The results of programs to improve the quality of care, are not encouraging. Perhaps these programs do not match with the problems experienced in family practice. This study aims to systematically explore how FPs perceive recognition, diagnosis and management of depressive and anxiety disorders.

**Methods:**

focus group discussions with FPs, qualitative analysis of transcriptions using thematic coding.

**Results:**

The FPs considered recognising, diagnosing and managing depressive- and anxiety disorders as an important task. They expressed serious doubts about the validity and usefulness of the DSM IV concept of depressive and anxiety disorders in family practice especially because of the high frequency of swift natural recovery. An important barrier was that many patients have difficulties in accepting the diagnosis and treatment with antidepressant drugs. FPs lacked guidance in the assessment of patients' burden. The FPs experienced they had too little time for patient education and counseling. The under capacity of specialised mental health care and its minimal collaboration with FPs were experienced as problematic. Valuable suggestions for solving the problems encountered were made

**Conclusion:**

Next to serious doubts regarding the diagnostic concept of depressive- and anxiety disorders a number of factors were identified which serve as barriers for suitablemental health care by FPs. These doubts and barriers should be taken into account in future research and in the design of interventions to improve mental health care in family practice.

## Background

Recognition and treatment of depressive disorders and anxiety disorders in family practice is not always in line with current medical standards. Intervention studies to improve the standard of care- focussing on education, dissemination and implementation of guidelines and use of screening instruments- are not particularly encouraging especially regarding patient outcome. Next to benefits of the programs we assumed that such interventions insufficiently match with the problems experienced by family physicians (FPs). Focus group discussions with FPs were held to explore and analyse the problems FPs encounter and to get sight the solutions they bring forward.

Depressive and anxiety disorders are the most common mental health problems in the population, with a prevalence of 4% respectively 5 – 10%, causing burden to patients and society [1,2]. Both disorders are often co morbid and form a common reason for consultation in family practice [2,3].

When compared to psychiatric interviews and current guidelines, underrecognition and sub-optimal treatment are reported; in just over half of patients with a major depressive disorder in family practice the diagnosis 'depression' is made, a quarter of them is prescribed an antidepressant subsequently which is, often in a low doses for a too short period of time [3-5]. For a number of patients better recognition and treatment can probably improve their health status [6]. However, there are indications that the labelling of patients' problems in terms of a disorder is not always important for successful management or relapse prevention[7]. Although there is a relative lack of primary care studies, this may indicate that there is still substantial room for improvement of patients' outcome in depression. The same might be true for anxiety disorders [8].

Recently, the effects of different interventions on the detection, management and outcome of depression and anxiety in family practice were assessed systematically [9,10]. Only interventions that combined strategies of clinician and patient education, nurse case management, enhanced support from specialist services and monitoring of drug compliance showed a positive effect but only of short duration [9,10]. We suppose that other barriers than knowledge and skills, such as in task perception, attitudes or interview-style, play a role in FPs recognition of depressive and anxiety disorders as well as patient factors and organisational barriers [11-13]. It is interesting that none of the studies included in the review, though all directed at the quality of care of depression, actually addressed problems FPs may encounter in recognising, diagnosing and treating depression. A qualitative approach seems the best method to analyse FPs' difficulties in this [14]. Some earlier qualitative studies reported problems of FPs in recognition, in differentiating between distress and depressive disorder and addressing depression as a medical/psychiatric disorder. They mainly focussed on depression, and did not address problems in management [15-21].

The aim of the present study was to systematically explore how FPs perceive recognition, diagnosis and management of depressive and anxiety disorders. In addition, we focussed on problems and barriers as experienced by FPs and listed the solutions the FPs proposed to get over these barriers.

## Methods

Focus group interviews are loosely structured interviews facilitating participants to offer general and specific information. It aims at exploring clinical experiences and beliefs and does not encourage the building of consensus. This makes it an appropriate qualitative method to explore complex problems while group interaction can trigger shared experiences [22-25]. For that reason focus group interviews were used in this study.

To obtain a wide range of experiences and to allow in-depth group discussions three groups from three different regions in the Netherlands were included in the study. Purposive sampling resulted in: (1) a long existing Continuous Medical Education (CME) group of FPs discussing topics on a monthly basis; (2) a group of FP-trainers of one of the eight residency training programs in the Netherlands and (3) a random group of FPs with their practices within 100 km of the Nijmegen university. Members of group 3 enrolled after 120 invitations had been sent to family physicians, 68 responded of whom 10 subscribed and 8 participated. To encourage participation, all FPs were paid (euro 125) for their attendance.

All participating FPs completed the Depression Attitude Questionnaire which measures the physician's attitude to depression and is considered as a valid and reliable measure of attitudes of FPs towards depression [25,26]. This is a visual analogue scale consisting of 20 questions with four components: treatment attitude, professional ease, depression malleability and depression identification [27].

After a brief introduction by the FP chairman a theme was introduced and each group member was given the opportunity to give his or her view. This individual round was followed by a group discussion. The meetings took place between November 2001 and April 2002, and lasted about 2.5 hours. Meetings were audio taped with consent of the participants and transcribed verbatim. The transcriptions were analysed independently by two raters (EvR, HvH) using thematic coding, with the help of ATLAS.ti, a qualitative data-analysis program [28,29] The results of individual analysis were compared and differences were settled by consensus [30]. Saturation of themes was reached after the third focus group and the data-collection was stopped.

## Results

### Participants

In total 23 family physicians (17 male, 6 female, age range: 41–59 years, all types of practices, urban, suburban and rural) participated in the study. For these characteristics the participants were comparable to Dutch FPs in general [31]. Participants' scores on the DAQ are presented in table 1. In general, the participants did not experience identification of depression as particularly problematic, held an optimistic view of its natural course and treatability, and felt relatively at ease in managing it.

**Table 1:** Mean scores of participants on four components of the depression attitude questionnaire (DAQ) range 0–100 mm

Component	Mean	SD (min-max)
Treatment attitude *High score = biochemical basis of depression, antidepressants useful, psychotherapy unsuccessful*	47.9	8.1 (31.3–65.8)

Professional ease *High score = uncomfortable managing depression, work is having going and not rewarding, psychotherapy should be left to a specialist*	63.8	10.2 (47.0–80.3)

Depression malleability *High score = pessimism towards depression, not amendable to change, is natural part of being old*	32.2	7.7 (15.8–47.5)

Depression identification *High score = difficulty distinguishing between depression from unhappiness, little help beyond FP*	41.1	14.6 (13.3–69.6)

### Tasks

Most participants considered recognition, diagnosis and management of depression and anxiety disorders an important part of their task, usually interesting but also rather time-consuming. A few participants doubted whether treatment should be a core-job for FPs. Most felt capable of managing most of their depressed or anxious patients.

### Conceptual doubts/Validity of diagnosis

A greater part of the participants expressed serious doubts of the validity of the diagnostic concept of depressive and anxiety disorders used in the DSM IV and practice guidelines [32,33]. They questioned whether depression and anxiety were always separate diagnostic entities or a syndrome or an arbitrary set of symptoms. They were reluctant to use these diagnostic labels, because a specific diagnosis had few consequences for treatment or prognosis. Particularly the demarcation between depressive disorders and anxiety disorders and other mental health problems was thought to be questionable, as the various features of these disorders were often, over longer periods of time, present in the same patient. Such fluctuation of symptoms- for example periods of anxiety or panic, followed by somatoform symptoms or depressive features- conflicted with the concept of distinct diagnostic entities. A more generic approach and superimposed symptom specific treatment would be helpful in the FPs' management of patients. Also, substantial differences in severity or burden between patients with the same diagnosis are seen by FPs. Nevertheless, some considered the criteria a useful diagnostic tool for diagnosing mentally distressed patients and they regarded a specific diagnosis helpful for guiding treatment. Attention to patients' non-verbal signs, particularly when observed over a longer period of time can be helpful in recognising depression and anxiety disorders, according to nearly all FPs.

#### Citations Conceptual doubts

*'I don not believe in those diagnoses, it are symptoms of other problems, for instance in youth, phase of life or social circumstances. Diagnosing an anxiety disorder is not useful at all....' *(FP 4, group B)

*' For me it is 'horse, trigger, bullet..., when I see patients with indistinct complaints I hand over a check list. If they score positive on 5 of the 9 items... they are depressed*.(FP 7, group C)

*'At a CME course I have learned to ask for the two core items of depression. In combination with my own appraisal I decide about the diagnosis.' *(FP 2, group C)

### Dealing with patients' preferences and patients' resistance

An important theme for the FPs was handling the preferences and resistances of patients. In the experience of the FPs patients with a mental health problem often presented themselves with physical (often vegetative) symptoms. This hampered diagnosis and further management of depressive or anxiety disorders. In particular as patients often deny the psycho-social nature of their symptoms. And patients seldom seek help with active reference to their mental health status. Difficulties in accepting the diagnosis 'depression' or 'anxiety disorder' as the explanation for their problems, anhedony, negative thoughts, feelings of shame a guilt and fear for stigmatisation, were in the eyes of the FPs important barriers for treatment while agreement about defining the problem is requisite. The FPs experienced that patients often had a strong resistance to psychopharmacological treatment, especially when prescribed for a longer period of time. This was related to fear for side effects and dependency. Patients often stopped taking their medication when symptoms had disappeared or diminished. The FPs felt also restricted in their treatment options due to patients' resistance towards referral to specialised mental health care professionals, because of emotional, social and financial barriers.

#### Citations Dealing with patients' preferences and resistance

*'patients only want to talk about the physical things, not about the mental ones. Often they are afraid to be qualified neurotic or depressed....' *(FP 2, group A)

*'Nearly all patients resist drug treatment; they think they have to overcome their problems all on their own and are afraid of side effects.... And when at last they are convinced to take antidepressants, they discontinue as soon as they feel better for a few days.' *(FP 3, group A)

### Distress or disorder?

The participants referred to the fact that, in their practice, they encountered often-psychological problems of a transient nature, as part of 'normal' life events. According to some, the distinction between such problems and a true psychiatric disorder was difficult. Therefore, most FPs were reluctant to label prematurely in diagnostic terms. For example, diagnosing major depressive disorder after only two weeks after presentation of the symptoms was perceived as far too quick. In this respect, the FPs expressed serious concerns of medicalising conditions they see as normal human distress. The assessment of the severity of the symptoms was perceived as crucial in deciding about the diagnosing a depressive disorder or anxiety disorder as described in the DSM IV and as important for deciding about treatment. Nevertheless, many FPs reported difficulties in how to assess the severity. FPs identified a number of patient groups in which recognition and diagnosis of depressive and anxiety disorders was particularly problematic: the elderly, patients with a different cultural background and patients with limited verbal skills. In patients with a chronic somatic-medical disease FPs noticed difficulties in interpreting the cause of physical symptoms. FPs expressed a deficiency in their knowledge of the specific anxiety disorders, and saw this deficiency as a potential cause of underdiagnosis in these patients. Continuity of care was usually seen as a helpful tool for diagnosis as it enabled them to monitor a patient's complaints and functioning over time. On the other hand some participants mentioned disadvantages of continuity of the doctor patient relation: getting too acquainted wit a patient may 'normalise' pathological mental distress and so, delay recognition of psychiatric disorders. Although the participants were positive about their communication skills in general, they experienced limited specific skills to cope and communicate with patients with mental health problems.

#### Citations Distressed or disorder?

*'many patients are distressed.... when I think it is serious I will talk it over....' *(FP 4, group A)

*'sometimes, you see a patient so often.... You become too familiar. When the patient visits a colleague, she easily recognises a depressed state of mind....'*.(FP 5, group A)

*'personally I have less rules of thumb for anxiety disorders... especially with the various types of this disorder.' *(FP 8, group C)

#### Antidepressants and beyond

The FPs expressed difficulties in deciding on best management. In their professional opinion there is a lack of knowledge of the natural history and long-term prognosis of (un)treated depressive and anxiety disorders. From that clinical experience FPs attributed a substantial placebo effect to antidepressant drugs. Persisting co-existing psychosocial problems or deprivation also limited the response to (antidepressant drug) treatment.

The FPs said to prescribe often relatively low standard dosages of serotonin reuptake inhibitors. They considered their knowledge of the different types within this group of drugs as rather limited and had concerns about how to discontinue antidepressants. In case of non-response they hesitated to increase dosage or to use other psychotropics.

The increased focus on antidepressants during a consultation, limited the application of other approaches such as psycho-education or counselling. FPs considered cognitive behavioural therapy (CBT) and problem solving therapy (PST) as valuable interventions, suitable in family practice, but experienced a deficit in skills to apply such techniques.

#### Citations Antidepressants and beyond

'I think we overvalue antidepressants, we use them too soon, much of their effect is natural recovery of the disorder' *(FP 5, group C)*

*'maybe I hesitate to diagnose a depression because of the long term treatment with antidepressant drugs....' *(FP 6, group B)

*'nowadays I spend so much time with talking about pills that there is barely time left for explaining the patients himself can do....' *(FP 3, group A)

### Conflicting demands and possibilities

In addition, a number of structural barriers were mentioned: a lack of time for detailed anamnesis and elaborate diagnostic procedures. This is reinforced because of limited reimbursement for additional time investment. The time available for a standard consultation was seen as too limited for CBT or PST. Time pressure also limited extensive psycho-education. Patients and FPs are confronted with long waiting lists for specialised mental health care. A major concern of the FPs was the non structural co-operation between family practice, primary care psychologists and specialised mental health care. Cooperation depended largely on personal relationships and experiences, only few mentioned more formal ways of cooperation like local or regional protocols or stepped care approaches

#### Citations Conflicting demands and possibilities

*'for removing a naevus surgically in 5 minutes I received an extra fee, talking 15 minutes with a anxious patient is not rewarded at all' *(FP 1, group A)

*'Finally, at the point the patient is convinced that referral is the best option.... we faced a waiting list of 5 months....' *(FP 4, group B)

You need a lot of endurance when trying to communicate with psychiatrist or psychologist. Getting them on the phone takes lots of time. (FP 4, group A)

### Needs and solutions

The group discussions did produce valuable solutions for the problems encountered. It emphasized the importance of using time as a diagnostic tool. FPs receive more then one opportunity to recognise a disorder. The approach of 'watchful waiting' when a disorder was suspected should receive more attention in clinical guidelines. Regarding management, patient education should be strengthened, aiming at empowering patients. FP -friendly psychometric tools for diagnosis and severity or mental burden are welcomed. Additional training on specific anxiety disorders, for communication skills to cope better with patients with mental health problems and for comprehensive psychotherapeutic techniques is needed. The FPs emphasized the need of a better co-operation with a limited number of specialised mental health care providers. Better financial rewards for the time-intensive treatment of depressive- and anxiety disorders and appointing practice nurse for systematic follow up of the patients was considered important.

## Discussion

The FPs valued recognising, diagnosing and managing depressive and anxiety disorders as important primary care tasks. However, many had strong reservations about the validity and usefulness of the DSM IV concepts of these disorders for family practice. Different diagnostic styles of the FPs were identified. With regard to diagnosis and management FPs expressed a mismatch between the recommendations in guidelines of a specific – often pharmacological approach and patients' preferences. Resistance against (long term use of) antidepressants and the fact that other psychosocial co-morbidity may overshadow or colour the features of depression and anxiety disorder, were seen as barriers for applying the guidelines. The management should focus more on patient empowerment than antidepressant prescription only. FPs seems to hesitate to use the diagnostic term depressive disorder or anxiety disorder while the fullfillment of these criteria imply a need for specific treatment. The argument of the need clear distinction between a diagnosis and need for treatment was also given from a theoretical point of view [34].

This study started out on the medical paradigm/model but during the study the usefulness of this model was disputed. For FPs 'patient context' or patient background variables were important in establishing mental health problems. One of the barriers in implementing evidence was that family physicians interpret evidence in an individual patients' context [35].

During the group discussions proposals were made to overcome the problems experienced. It was noteworthy that the FPs touched upon a number of unresolved issues in the medical literature: the effectiveness of antidepressants in mild depressive disorders and the management of co-morbid psychiatric disorders [36]. This underlines the need to take practical clinical experience from primary care into account in the design of further research on mental health problems.

Although this study provided important new information, a number of limitations of its design should be taken into account. The explorative design with a limited number of FPs may hamper the extrapolation of the results to all FPs. The method of the focus group discussions worked quite well and yielded problems the participants experienced in all domains of their clinical practice of depressive- and anxiety disorders. Rigour was enhanced using the DAQ as an instrument for triangulation. The scores on the DAQ are in line with previously reported studies, also indicating that the participants of this study represented the variation in FPs attitudes towards mental health problems [25,26]. Unfortunately specific Dutch reference data concerning the DAQ are lacking. In the Netherlands most health problems are treated in primary care and FPs are serving as a 'gate keeper' for secondary care. As many other countries have comparable health care systems and also a mix of private and public funding the results of this study generalise to other countries as well.

The serious conceptual doubts have not been presented earlier, but some barriers had been reported earlier in a review, which was based on epidemiological data and theoretical considerations rather then on the experience of FPs[11,37,38]. The FPs' opinions about the extremely short 2 week period of the presence symptoms to diagnose a depressive disorder is supported by epidemiological data [39]. As well as a high recovery rate of depressive disorders within three months without a formal intervention [40]. Most qualitative studies published recently, did examine the FPs experience in recognizing depression [15-21]. Recently the patient perspective on talking with doctors about depression was published [41]. Recognition and management of anxiety disorders were not studied earlier [15-21]. Only a Swedish study reported on the management of depressive disorders, mainly on pharmacological treatment [21]. The GPs in our study reported considerable reservations regarding antidepressant drugs, felt unskilled to offer other specific treatment modalities (like problem solving treatment) and experienced difficulties in cooperation with specialized mental health care. These difficulties are reflected in the relatively high score on the DAQ subscale professional ease.

A study on British FPs did not report time pressure which was emphasized in this study as well as by British patients [18]. The difficulties in discriminating between psychological distress and a psychiatric disorder were reported earlier by Swedish FPs. They also modified the concept of depression with different causes and expressed reservations of the increase in antidepressant prescribing [21]. It also emphasised the relevance of non-verbal signs and pre-existing knowledge of FPs. In accordance with our results the collaboration with psychiatry consultants was perceived as unsatisfactory [17]. The difficulties in management depressive disorders in patients with persisting psychosocial problems as reported by the FPs was described earlier in a study with FP working in socio-economically deprived areas [16].

## Conclusion

This study confirmed the FPs' substantial professional role in the diagnosis and management of depression and anxiety. The FPs identified a number of factors that hamper the performance of this role, some of these were not reported earlier. These factors refer to insufficient understanding of the natural history, and course over time, of mental health problems. It stresses the importance of a primary care research agenda of mental health problems focussing on those factors. It should form an integral part of the further improvement of mental health care. We recommend to pay more attention to patient education/psycho education, patient activation, self-management programs in family practice, the need for user-friendly psychometric tools for assessment and monitoring. For instance the use of the PHQ-9 or the Beck Depression Inventory. The instruments can also be used for monitoring the course of the disorder when using a watchfull waiting strategy or to evaluate treatment effects. Some of the approaches mentioned above can be provided by FPs, other by (community) mental health nurses working in family practice.

Development of an effective generic approach for the management of various mental health problems in family practice and additional training for comprehensive psychotherapeutic techniques is a priority. The FPs emphasized the need of a better co-operation with specialised mental health care providers. Various collaborative care models are developed, seem effective and can be used in different health care models.

In addition, the barriers and solutions should be taken into account in the design of primary care based interventions on recognition and management depressive- and anxiety disorders. This may result in better patient outcome and provision of cost effective care.

## Competing interests

The authors declare that they have no competing interests.

## Authors' contributions

EvR and HvH: have made substantial contributions to conception and design, or acquisition of data, or analysis and interpretation of data;

EvR, HvH, EvdL, FZ and CvW have been involved in drafting the manuscript or revising it critically for important intellectual content and have given final approval of the version to be published.

## Pre-publication history

The pre-publication history for this paper can be accessed here:


